# New investigations of the Hjortspring boat: Dating and analysis of the cordage and caulking materials used in a pre-Roman iron age plank boat

**DOI:** 10.1371/journal.pone.0336965

**Published:** 2025-12-10

**Authors:** Mikael Fauvelle, Boel Bengtsson, Olof Pipping, Mikkel Hollmann, Martin Nordvig Mortensen, Peter Toft, Sahel Ganji, Ashely Green, Christian Horn, Stephen Hall, Flemming Kaul, Johan Ling

**Affiliations:** 1 Lund University, Department of Archaeology and Ancient History, Lund, Sweden; 2 University of Gothenburg, Department of Historical Studies, Gothenburg, Sweden; 3 Independent Researcher, Alingsås, Sweden; 4 Ringkøbing Fjord Museer, Rinkøbing, Denmark; 5 National Museum of Denmark, Copenhagen, Denmark; 6 Lund University, Division of Solid Mechanics, Department of Construction Sciences, Lund, Sweden; Israel Antiquities Authority, ISRAEL

## Abstract

The Hjortspring boat is the only intact example of a prehistoric sewn plank boat ever found in Scandinavia. Built from lime wood planks lashed together with cordage, the boat represents the maritime technology used by some of Northern Europe’s earliest seafarers. This article reports new analysis of the cordage and caulking material used in the construction of the Hjortspring boat. We provide the first ever direct date for the boat based on materials from the original excavation finds, with lime bast cordage from the boat carbon dated to between 381 and 161 BCE. We report the results of GC-MS analysis of the material used to caulk the boat, which shows that it was made from a mixture of animal fat and pine pitch. We argue that the use of pine pitch in the boat’s construction indicates that the boat was not built on the Jutland peninsula and instead came from a region with more abundant pine forests. Based on the dispersal of pine forests in Northern Europe during the first millennium BCE, we propose the Baltic Sea Region east of Rügen and Scania as a likely source for the boat and its crew. We also analyze intact cordage fragments and imprints of cordage on caulking material in order to describe the sewing and rope-making techniques that were used to construct the boat. Finally, we report on the discovery of a partial human fingerprint found on a fragment of caulking material. This remarkable fingerprint provides a direct link to the ancient seafarers who used this boat. Together, these results shed new light on methods and materials used to build Scandinavia’s first plank boats and raise new questions regarding our understanding of early maritime societies in Northern Europe.

## Introduction

**Fig 1:**
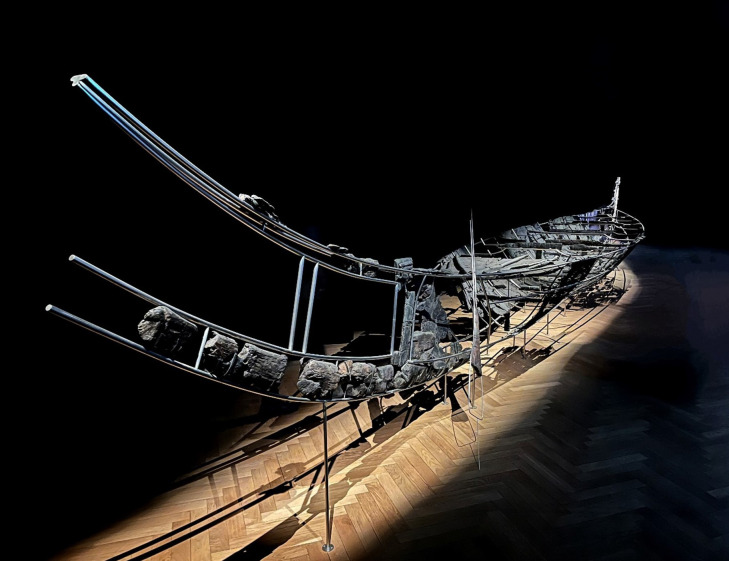
The Hjortspring boat as currently displayed at the National Museum of Denmark. Photo by Boel Bengtsson.

**Fig 2:**
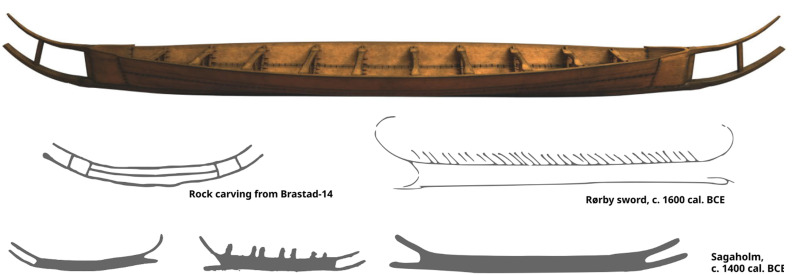
Comparison of Hjortspring boat (Above, 3D model by Richard Potter) with securely dated Bronze Age art (Rørby sword and Sagaholm rock art) as well as an example of early Iron Age art from Brastad. Thousands of other examples of Bronze Age boat depictions exist. Note the continuity in form and design evident in these different boat depictions.

Over one hundred years after it was first discovered, the Hjortspring boat remains one of the most important finds of an ancient watercraft ever made in Northern Europe (Fig 1). As the only intact example ever discovered of a prehistoric plank boat, the Hjortspring find represents a unique opportunity to study some of the earliest plank-boatbuilding to have developed in Scandinavia. While the artifacts found in the boat have long indicated that the find dates to the pre-Roman Iron Age, strong stylistic parallels between the boat’s form and depictions of boats on Bronze Age rock art have suggested that the Hjortspring boat may represent a boatbuilding tradition that started during the Bronze Age (Fig 2). New studies of the Hjortspring boat thus give the opportunity to shed light on early seafaring from both the Iron and Bronze Ages.

The Hjortspring boat was discovered in 1880s during peat digging in the Hjortspring bog on the island of Als off the southeastern corner of Jutland, Denmark [1]. Formal investigations of the bog and boat find started in 1920 following the reunification of southern Jutland with Denmark [2]. The boat was excavated by Gustav Rosenberg between 1921 and 1922 and about 40% of the original boat was recovered from the bog, allowing for a full reconstruction of the boats form [3,4]. Enough iron spearpoints and shields to outfit a military band of around 80 warriors were deposited together with the boat – far more individuals than could have fit inside the boat itself. Based on these finds it has been suggested that a band from an unknown location traveling in up to four Hjortspring style boats attacked the island of Als and was defeated, with the victors depositing the weapons of their vanquished foes together with one of their boats into the bog to give thanks for the victory [5]. After excavation the boat was conserved and has been on display in the National Museum of Denmark since 1937 [6]. A full-scale modern reproduction of the Hjortpsring boat, called the *Tilia Alsie*, was constructed in the early 2000s and generated a great deal of information regarding the vessel’s construction techniques and technical capabilities [1,7,8].

The design of the Hjortspring boat shows strong similarities to depictions of boats in Bronze Age rock art (Fig 2). While the standard Bronze Age rock carving boat is of asymmetrical design (during the Late Bronze Age boats display highly raised lower horn projections in the bow), upper and lower horn projections of the Early Iron Age are of a more symmetrical and much less elaborate design [9,10]. Even though these differences in the boat profile are easily discernible, the rock carving evidence demonstrates a continuity in the boat building tradition during Latest Bronze Age/Early Pre-Roman Iron Age. Transitional or hybrid boat shapes have been recorded, for instance at Tanum, Bohuslän, Western Sweden, showing a ‘gradual symmetrization’ and a lowering of the horn projections in the stem and stern [10]. On the Baltic Island of Bornholm, the Madsebakke rock carving site has yielded a splendid example, where the transitional process can be observed, from the asymmetrical silhouette of the boats of the Bronze Age towards the more symmetrical shapes of Pre-Roman Iron Age [9,11]. Thus, the basic structure/profile of the Hjortspring boat can be followed back in time to the Bronze Age, throughout the period of which the shape of the projecting horns in stem and stern continued to change over time, providing a means to date them.

The dating of the Hjortspring boat has long been a subject of scholarly debate. Diagnostic finds within the boat place the find within the pre-Roman Iron Age, but there is some disagreement as to whether the find best matches a date to the beginning, middle, or end of this period (e.g., 4^th^, 3^rd^, or 2^nd^ century BCE) [12–15]. As all wood finds from the boat were immediately and thoroughly conserved with alum and later with polyethylene glycol (PEG), it is impossible to obtain an accurate C14 date from the boat itself [4]. In 1987 a team from the National Museum of Denmark returned to the excavation site and opened new test pits in order to search for any remaining unconserved wood [2]. Wood fragments found during this excavation were carbon dated to 2240 ± 50 BP (390−210 BC with ± 1 standard deviation) and 2290 ± 70 BP (400−260 BC with ± 1 standard deviation) [5]. Based on these finds the boat seems most likely to date to either the 4^th^ or 3^rd^ centuries BCE.

The question of the origins of the Hjortspring boat and its crew has also provoked considerable scholarly debate [5,12,16]. As the boat is believed to have been used by invaders who attacked the island of Als it is widely acknowledged that it likely came from elsewhere. Where exactly the attack originated from, however, has been a long-standing mystery. Proposed source locations include nearby regions such as the Jutland Peninsula or the island of Funen, as well as more distant locations across the Danish Isles and coastal northern Europe (Kaul 2003: 176–177). The weapons found on the boat have parallels across northern Europe, making it difficult to pin down a source location. Wooden containers from the boat give slightly more information, with parallels identified to objects from Denmark, northern Germany, southern Sweden, Bornholm, and Gotland (Kaul 2003: 160). Based on the typological analysis of the find material alone, however, pinning down the exact origin point of the ship has proved elusive for previous researchers.

The present study reports the analysis of a collection of previously unreported caulking and cordage fragments that were collected during the original excavation of the Hjortspring boat and have since been curated at the National Museum of Denmark. In addition to C14 dating an unconserved fragment of cordage from the original excavation, we also analysed the caulking materials used on the ship using gas chromatography and mass spectrometry (GC-MS). Our analysis indicates that the caulking material was made with pine pitch, providing a new line of evidence on the ship’s possible provenance. During the inspecting of these caulking materials, a partial fingerprint was found on a piece of caulking resin, which is also reported in this paper. A careful analysis of cordage fragments and imprints of cordage on caulking resins also provided new insights into the sewing and rope-making techniques that were used to construct the boat. Together, these new analyses confirm the 4^th^ or 3^rd^ century BCE date of the boat and provide new insights into the construction, use, and provenance of the vessel.

## Materials and analysis

**Fig 3:**
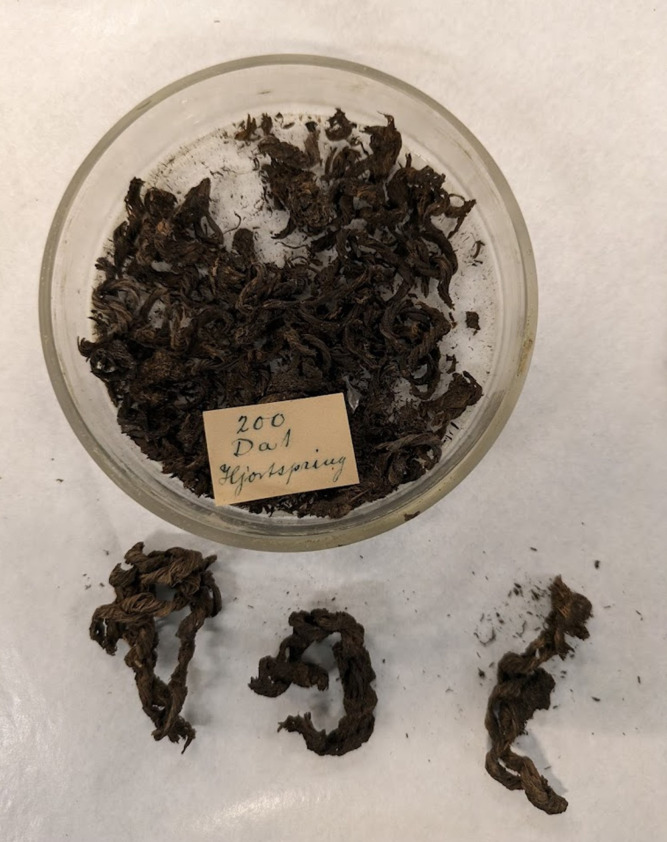
Small dish of cordage from original excavation of the Hjortspring boat. Three of the larger cordage fragments have been removed and can be seen at the bottom of the photo. This material was radiocarbon dated to get a direct date for the use of the boat.

Extensive studies of the Hjortspring boat were carried out after its excavation and again during the reconstruction of the *Tilia Alsie* full-sized boat reconstruction [1,7,8]. While most of the original excavation material was conserved with alum and PEG, some collections of unconserved and un-studied material still remain. This includes a collection of caulking materials that were sent to the Viking Ship Museum in Roskilde and then returned to the National Museum during the late 1980s. This collection then sat uninvestigated for several decades until they were re-examined by the authors of this paper in 2024. While investigating this caulking material a small container of unconserved cordage from the original excavation was also discovered stored in the same collection (Fig 3). Since these materials had not been studied in over four decades, it was determined that they could benefit from new analysis using contemporary expertise and current scientific methods. In the following discussion we present the results of our analysis of these materials. We focus first on the cordage and then on the caulking resins, presenting both methods and results for each material type in turn. This is followed by a discussion of the implications of our results for understanding Bronze and Iron Age seafaring in Scandinavia.

### Cordage: Scanning and dating

The Hjortspring boat is a sewn plank boat, meaning that it was constructed by lashing together planks with cordage instead of fastening them with nails. Cordage was therefore a critical component of the ship’s construction. Previous discussions of the cordage material used in the ships construction were largely based on the imprints left by cordage on the caulking material [6], as well as a brief mention of a few “samples of strings made of bast” [17]. It is unknown if the cordage described in this paper are the same fragments mentioned by Valbjørn and Rasmussen as they provide no detailed description of those samples [17]. As the labeling on present collection was written with an ink pen in an antiquated cursive script, we suspect that they are in fact different samples that had not been inspected since the excavation of the boat.

**Fig 4:**
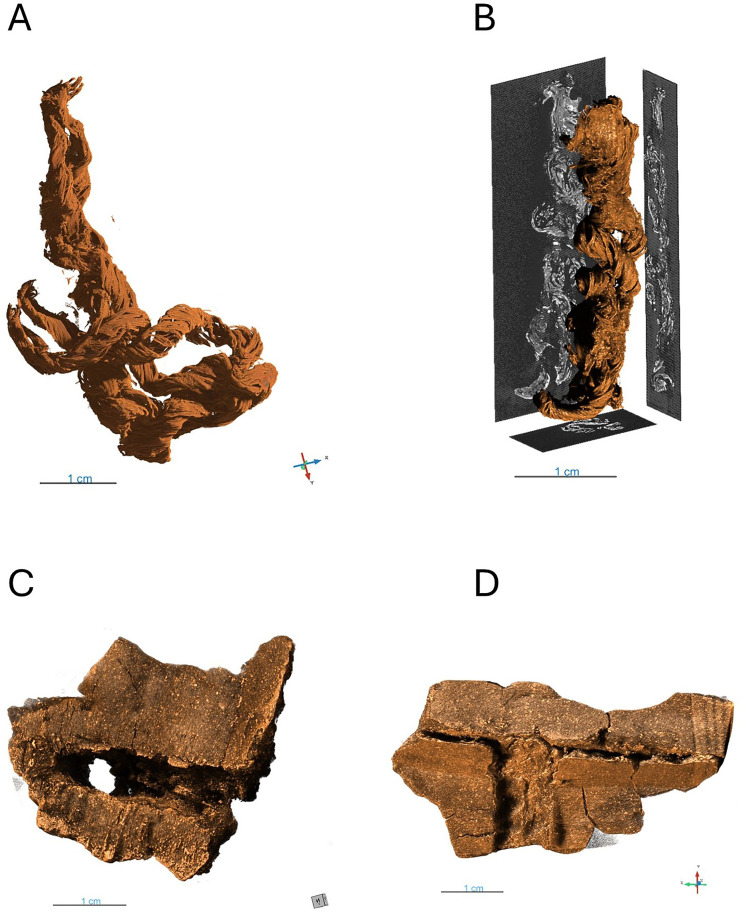
3D renderings of X-ray tomography scans of cordage and caulking materials from the Hjortspring boat. A: 3D rendering of two ply cordage fragment. B: 3D rendering of 2 ply cordage fragment showing x-ray slices. C. 3D rendering of caulking material showing cordage imprint and hole. D. 3D rendering of caulking material showing cordage imprint.

**Fig 5:**
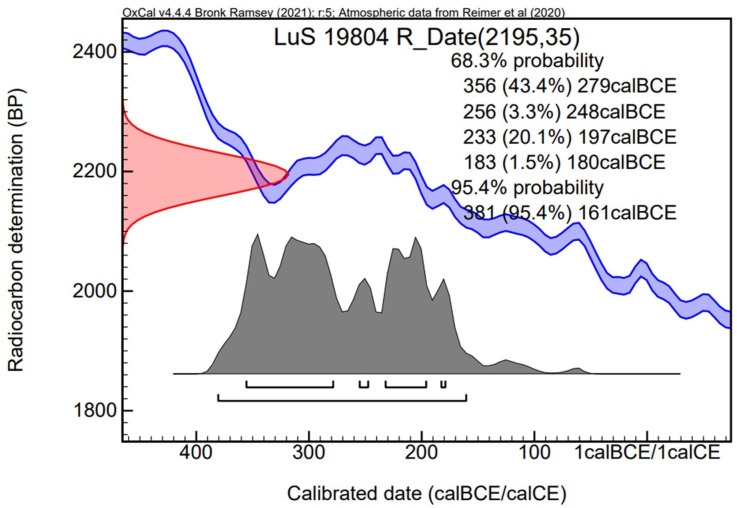
Calibration curve and radiocarbon date determinations for lime bast cordage from original excavation of Hjortspring boat. Note plateau in calibration curve between circa 350 and 200 CE.

The cordage fragments presented in this paper vary in size from 3.5 cm to less than a millimeter. In order to digitally conserve and better understand these cordage fragments we produced high-resolution 3D models of a selection of the fragments (Fig 4). The X-ray tomography was performed at the 4D imaging Lab at Lund University using an RXSolutions EasyTom150 (See S1 File for additional experimental information). A small fragment of cordage was carbon dated and returned a date of 2195 ± 35 BP, which was calibrated using OxCal online to dates of 381−161 cal BCE (2 sigma, or 95.4% probability) or 356−180 cal BCE (1 sigma, or 68.3% probability) (Fig 5). The relatively flat calibration curve during the 4^th^ and 3^rd^ centuries BCE unfortunately prevents a more precise estimation. This is the first direct date of material from the original Hjortspring excavation material. The dating of the Hjortspring cordage confirms the Pre-Roman Iron Age date of the vessel and corresponds well with previous attempts to date the boat (**see discussion**).

### Cordage: Technical analysis and experimental reconstruction

The rediscovery of intact fragments of cordage from the Hjortspring boat allowed us to study the technical capacities of the cordage and the production techniques used to make it. The cordage fragments consist of two strands, spun directly from raw material, which are laid together to form a rope-like cordage. As the cordage in this find material is thin (circa 3–4 mm), it is technically classified as string, rather than rope. The string fragments seem to be very loosely laid, with the strands wound around each other with a lot of air between the strands. This is probably due to drying post-excavation. Additionally, the strands seem to be spun from a thread-like material, but this is most likely the result of degradation of the material. Post excavation drying shrunk the strands as well as contracting them, creating two separate spirals with a shorter lay than when it was in its original state. Luckily, the inherent character of the spin in the strand is preserved and from this the original string can be recreated. The strands in the string are S-spun, which are laid into a Z-lay. Visual inspection of the string using a 10x magnifying loop strongly suggests that it is made from lime bast.

Based on our inspection of the strands they seem to have been spun with a spinning hook, a very simple yet effective tool. Spinning hooks are made from twigs and form a hook with which to spin the material and store the spun strand when it is too long to handle. To lay the string, the end of the strand would have been hitched to a fixed point and stretched out. Half the strand is wound on a pin (a laying stick) and the strand bent around at the mid-point. The roper lays the strand around the stretched part towards the fixed point, while a helper handles the laying stick, one turn at the time. The roper lays the strand tightly, while making sure the balance in the string is upheld. The spin in the strand (the hardness of it) must be balanced against the hardness of the lay. While earlier reconstruction work on the Hjortspring boat mostly used a reel and ropewalk, the method described above is more suitable for bast cordage making.

The strands in the string have a long spin (i.e., few turns per length), which also has implications for the construction of the ship. These strands have been given a long lay when producing a two-stranded string. A long lay would be perceived as loose and pliable which would be an asset in the context of boat building and repair, as it is easier to reeve through holes and tighten than a more knobbly short lay. For more information on the laying and reeving of rope we refer readers to works by Ingeborn, Modéer, and Schjølberg [18–20]. The quality of the material indicated in the fragments points to very well-prepared material of a fineness allowing for a nicely laid, even and strong string. The retting of the material would also have been very important as the retting process must be interrupted at the moment the bast layers are well separated but not degraded. This would provide ample amount of good material, both fine and coarse.

**Fig 6:**
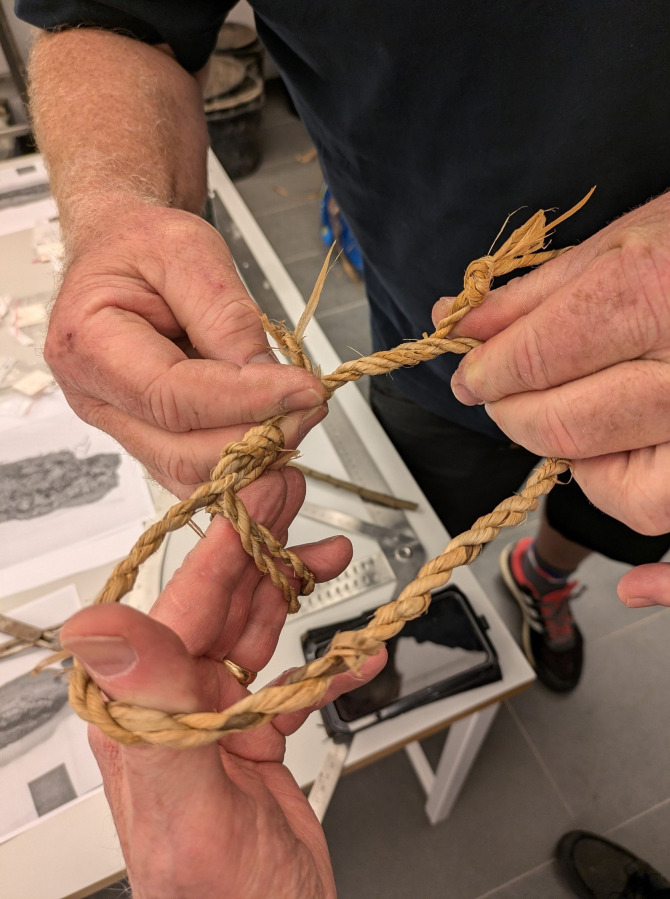
Depiction of our experimental reconstruction of lime bast cordage and hitch knot. This reconstruction was made by Mikkel Hollmann and Olof Pipping using a spinning hook. Note that some sections are two ply while others are four ply. Photo by Mikael Fauvelle.

Based on our observations of the intact cordage fragments we were able to reconstruct a 1,5 meter segment of string similar to that which would have been used in the Hjortspring boat (Fig 6). Through our reconstruction we were able to solve a question that was raised during the reconstruction of the Tilia Alsie in the 1990s; why some imprints of cordage in caulking material seem to have different strand counts [7]. To replicate the knots suggested in Crumlin-Pedersen & Trakadas (2003) that would have lashed together boards on the boat, we found that by reeving a string two times in holes in the planks, the string then worked tight and locked with a single hitch, works very well if the hitch is taken with the lay of the string, which will also create the impression of a four-laid string at the hitch. Lengthening of the string would have apparently been made by laying in a new piece of string into the end, which will create an impression of a short four-laid string, as is evident in the caulking. We found that by combining cords in this way we could securely connect strings to create longer lengths. This could also have been very useful for construction repairs as it would allow short sections of string to be incorporated without bulky knots into the sewing of the boat instead of requiring long ship-length series of cordage. It also explains why we see both 2-strand and 4-strand imprints of cordage in caulking impressions while all the fragments we have found contain only 2 strands.

Taken together, the results of our analysis of the Hjortspring cordage illustrate the skill and sophistication of ancient Scandinavian boatbuilding techniques. It is clear that the cordage found in the boat was made by highly skilled craftspeople who were well versed in what must have been a long-standing boat building tradition. Due to the fact that the cordage fragments were untarred, it is possible they were kept on the boat for potential repairs. Such cordage could have been spliced into existing lines in the manner identified in our experimental trials. It is likely that both caulking material and cordage were kept on the ship in order to conduct repairs while at sea.

### Caulking: Chemical analysis and provenance

Several hundred fragments of caulking material were excavated together with the Hjortspring boat, of which only a small number had previously been described (e.g., 1). Caulking materials have imprints of boat design elements including cordage, knots, and plank seams, all of which can be extremely useful for understanding the construction of the boat. While most of these imprints are positive (design elements pushed into the tar), a few contain negative imprints, which we interpret as repair material applied to damaged or removed caulking. Here we report the first ever analysis of the Hjortspring caulking samples using GC-MS analysis, as well as the identification of a previously unknown fingerprint found on caulking material from the vessel.

**Fig 7:**
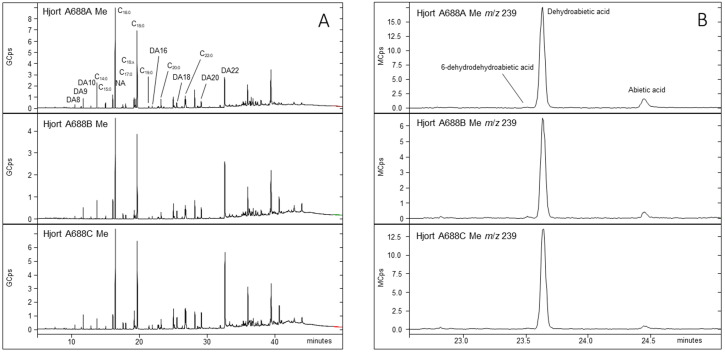
Chromatograms recorded on methylated extracts of the Hjortspring boat caulking samples A688A, A688B and A688C. TIC (total ion count) from the full scans are shown in (A) and a plot of the ion m/z 239 is shown in (B). DA8: Dicarboxylic acid with 8 carbon, C_14:0_: *Fatty acid with 14 carbon and 0 double bonds, C*_18:X_: *Several unsaturated C18-fatty acids, NA: 18-norabietane.*

Gas chromatography – mass spectrometry (GC-MS) analysis was carried out on TMS (trimethylsilyl) derivatives of the three samples from the Hjortspring boat caulking (See S1 File for experimental procedures). GC-MS spectra showed peaks that could be assigned to glycerol, palmitic acid and stearic acid, possibly components from a tri-glyceride which could possibly be lard. Small signals were detected as well, corresponding to 8-Isopropyl-1,3-dimethylphenanthrene, methyl pimaran-18-oate and dehydroabietic acid trimethylsilyl ester. These compounds are related to abietic acid, which is common in coniferous resin and -tar [21]. A methylation was carried out as well on another portion of each of the three samples. GC-MS recordings (Fig 7, panel A) showed strong signals corresponding to fatty acids (C_14:0–20:0_, C_22:0_, C_18:X_) and dicarboxylates (DA8–10, DA16, DA 18, DA20, DA22) which is also consistent with fat/lard maybe combined with plant wax from the soil which appeared to have been in contact with the caulking. GC-MS analysis of this methylated extract also showed 18-norabietane, 6-dehydrodehydroabietic acid, dehydroabietic acid and abietic acid, the latter three are observed in the plot of *m*/*z* 239 shown in Fig 7, panel B.

The mass spectrum of a TMS derivatized betulin reference, could not be convincingly matched to any peak in the chromatograms from the Hjortspring samples. Since betulin is characteristic in birch trees and in products hereof such as tar or pitch, the presence of birch tar in the caulking mixture on the Hjortspring boat seems unlikely. Instead, this analysis indicates that coniferous tar, probably pine, and fat were constituents of the caulking material in the Hjortspring boat.

### Caulking: The Hjortspring fingerprint

**Fig 8:**
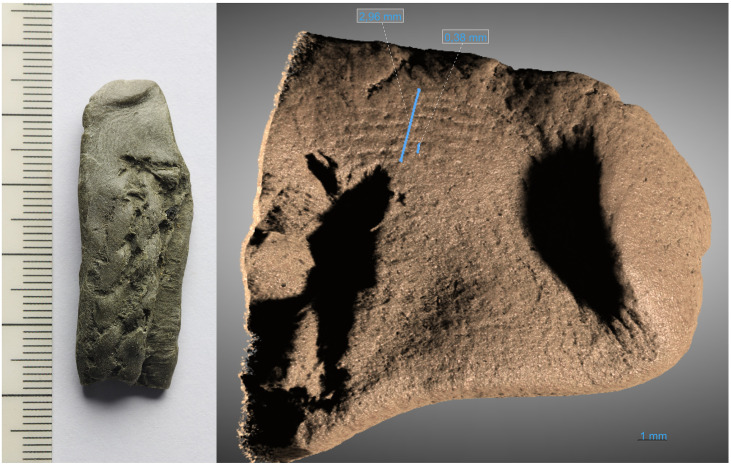
Photo of caulking fragment showing fingerprint on the left and high-resolution x-ray tomography scan of fingerprint region on the right. Photography by Erik Johansson, 3D model by Sahel Ganji.

During our inspection of the Hjortspring caulking materials we identified a partial fingerprint on a small caulking fragment (Fig 8). This was confirmed to be a partial fingerprint through correspondence with Julie Hruby, a leading expert on archaeological fingerprints (Personal communication 2024). The fingerprint is on the tip of a caulking fragment with a positive impression of cordage and is interpreted by the authors as being a fragment from caulking repair which was applied to the boat after its manufacture. A total of 7 ridges are visible over a distance of 2,96 mm, with an average ridge width of around 0,4 mm. These ridge sizes fall within average distributions for both adult male and females as well as for juvenile adults, making it difficult to say much about the individual who produced the print. The most likely interpretation, however, is that it was made during repairs by one of the crew members on the boat itself, providing a direct link to the seafarers of the ancient vessel.

## Discussion

**Fig 9:**
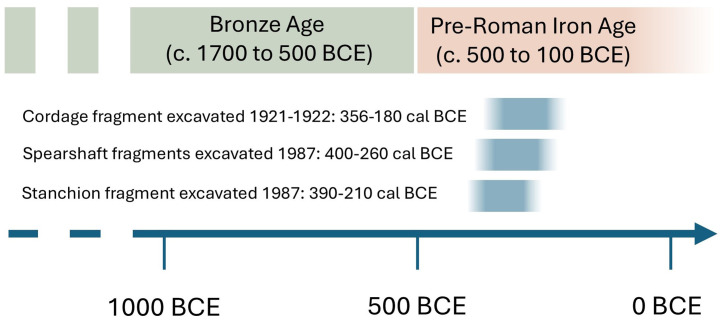
Schematic of carbon dates from the Hjortspring boat showing 1 sigma date ranges. From these dates we can estimate that the boat was most likely in use during the 4^th^ century BCE.

The results of our new analysis of the caulking and cordage materials from the Hjortspring find allow us to address several old debates regarding the dating and provenance of the boat. The new C14 date of the cordage used in the indicates that the boat was most likely in use during the 4^th^ or early 3^rd^ centuries BCE. Previous C14 dates from wood fragments excavated from the Hjortspring site during the 1980s also dated to the 4^th^ and 3^rd^ centuries BCE, yet it has been argued that they could be subject to an “old wood” effect as the felling of the trees used on the boat’s construction could substantially pre-date the burial of the boat itself. Cordage is unlikely to suffer from this problem, as cordage used on a boat is likely to be replaced over the course of a few years – a short period of time for C14 dating. The fact that our dates for the boat’s cordage overlap previous dates from the boat strongly suggests that the Hjortspring boat was used during the 4^th^ and possibly the beginning of the 3^rd^ centuries BCE (Fig 9). These results indicate that the boat dates to the first half of the Pre-Roman Iron Age and can hopefully settle long-standing disputes over the rough chronology of the ship’s construction.

**Fig 10:**
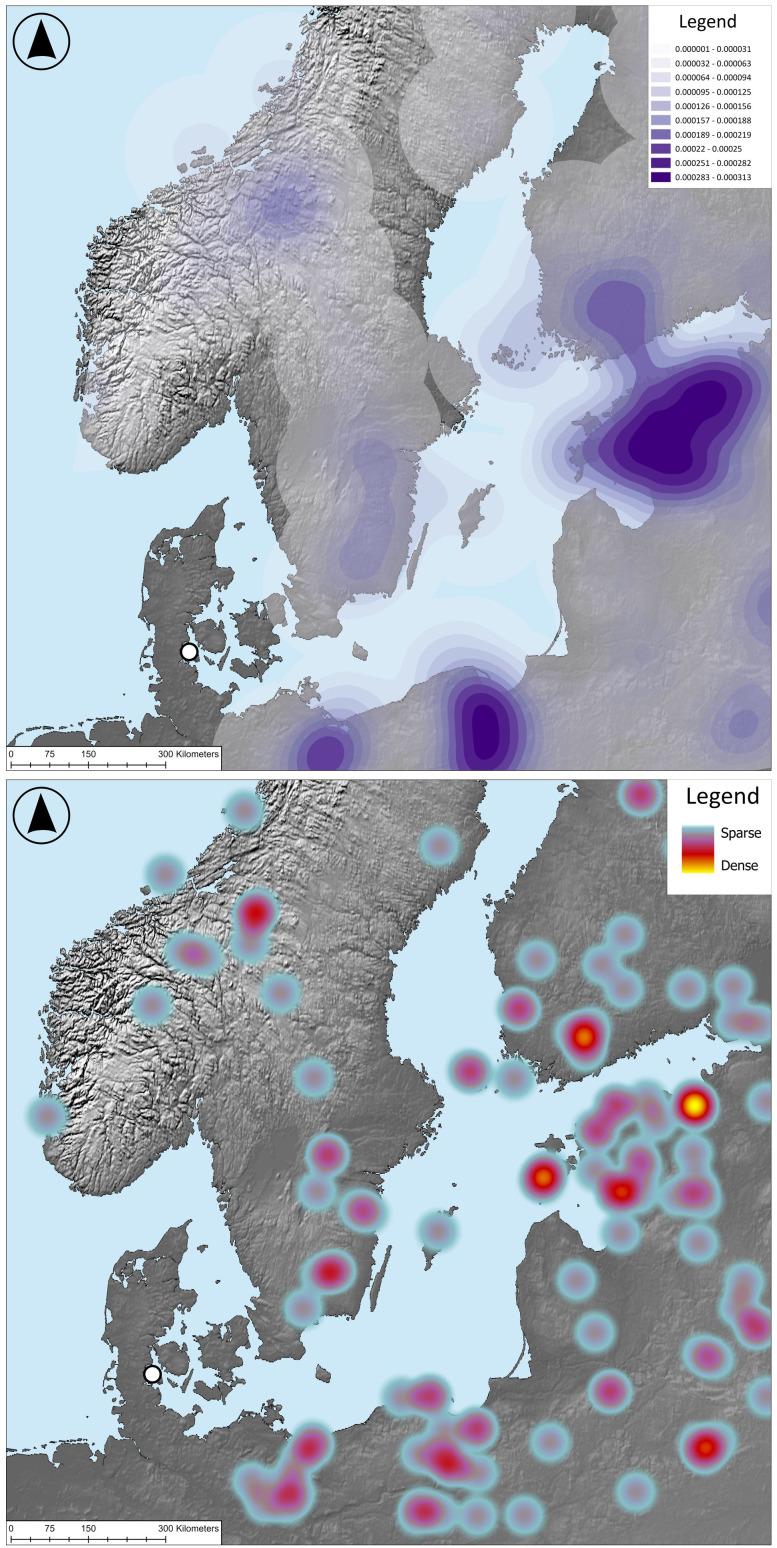
Two maps showing dispersal of pine trees in Scandinavia and the Baltic Sea Region during the first millennium BCE based on Pinus sp. pollen data. Map on top shows Kernel Density Estimate and map on bottom shows a heat map based on the distribution of sites with a minimum of 25% abundance of pine pollen (From the European Pollen Database [24], *map by Christian Horn). Hjortspring find site is represented by a white circle. See also work by Githumbi and colleagues* [25] *for maps of coniferous tree coverage during the same time period using different data sets. Note the lack of pine tree coverage in Denmark and northwestern Germany, both of which were cleared of coniferous forests during the Neolithic Period. Basemaps were made with Natural Earth. Free vector and raster map data @ naturalearthdata.com.*

The identification of pine pitch in the ship’s caulking material allows us to address the question of the ship’s provenance. While Denmark had numerous pine forests during the early Holocene, these had almost completely disappeared by the start of the Neolithic following large scale land-clearance for agriculture. Both pine pollen counts and microfossils of pine show that pine trees would have been very rare on the Danish landscape after around 6000 BCE [22–25]. The shores of the Baltic Sea east of Rügen and Scania, on the other hand, had abundant pine forests during the first millennium BCE [24,25] (Fig 10). While we cannot rule that pine pitch could have been traded from this region to the makers of the Hjortspring boat, it seems more likely that shipbuilders in areas lacking in Pine forests would have used other local materials such as birch tar (for regions with deciduous forests) or linseed oil (for open areas cleared for agriculture) [26]. We therefore suggest that it is likely that the Hjortspring boat and its crew came from a region with abundant pine forest coverage. According to this analysis, the most likely source regions for the Hjortspring boat would therefore have been areas bordering the Baltic Sea such as Blekinge, Gotland, northern Poland, or Bornholm.

**Fig 11:**
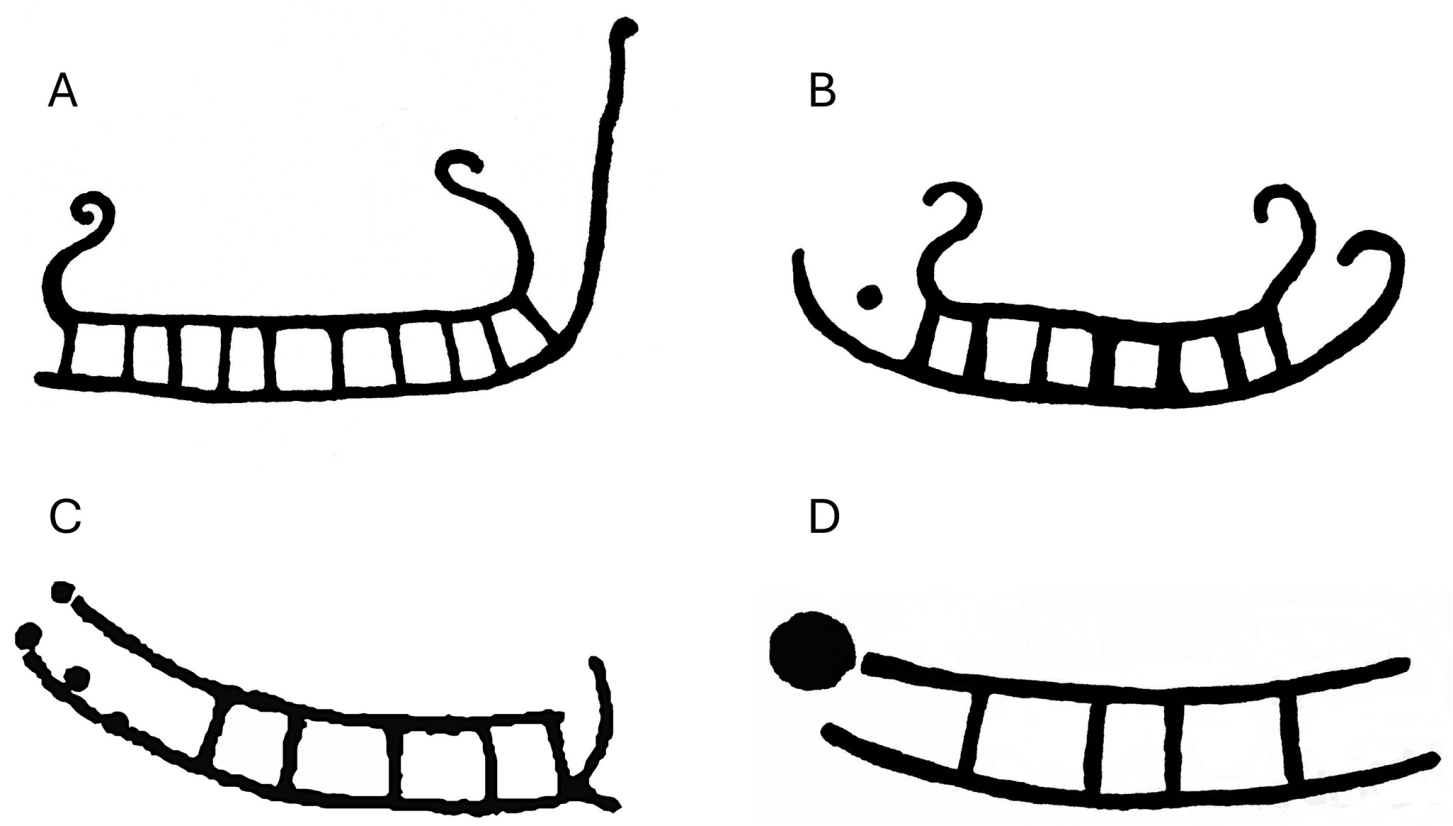
Rock carvings from North Bornholm showing changes in boat design/silhouette from Late Bronze Age through the Pre-Roman Iron Age. Note the gradual nature of these changes, indicating a continuity of boat-building traditions. In chronological order these rock carvings are A. Madsebakke ship 1: Typical asymmetrical Late Bronze Age ship with high lower horn projection. B. Madsebakke ship 5: Increasingly symmetrical boat from the very end of the Bronze Age, C. Madsebakke ship 11: A transitional form showing elements of both Iron Age and Bronze Age boat design. D. Rock art from Hammersholm showing a Hjortspring-style Pre-Roman Iron Age boat.

The boat images of the rock carvings demonstrate that the symmetrical boats of obvious Hjortspring-like shapes were in use during the Early Pre-Roman Iron Age in larger areas of Scandinavia, even in North Trøndelag, Central Norway, as well as in South Norway, and in Bohuslän, Western Sweden [27–29]. Seen from the rock carving evidence alone, an invading fleet could come from any region, where such ships or boats were depicted. It has previously been suggested that insufficient quantities of lime trees grew in the northern reaches of the Baltic to support the construction of the Hjortspring boat (Randsborg 1995). Symmetrical Hjortspring type boat carvings, however, are well documented on the Baltic Island of Bornholm [9,11]. Rock art from Bornholm demonstrates the changes in ship design from the Late Bronze Age to the Pre-Roman Iron Age and include direct typological matches to the design of the Hjortpsring boat itself (Fig 11). This line of evidence therefore supports the hypothesis that the Baltic Sea Region may have been the source location for the Hjortspring boat.

The suggestion of the Baltic Sea east of east of Rügen and Scania as a source location for the Hjortspring boat should be seen as a proposed hypothesis for further testing rather than a secure determination of the vessel’s provenance. It is possible that pitch could have been traded over considerable distances, as it was during the Viking and Medieval periods [30,31]. Other regions, such as Bohuslän in western Sweden, might also be possible source locations due to proximity to large inland forests [32]. Additionally, there may have been isolated pine trees that could have been exploited to limited degrees for resin even in regions mostly devoid of coniferous trees. Both of these factors present limitations to our suggested provenance for the boat based on the composition of its caulking material. Nonetheless, we suggest that ancient boat builders would have used the material they had nearby in the greatest abundance when constructing vessels. In most regions of the Danish Archipelago or northern Germany this would have meant a mix of linseed oil and tallow, which is what was used to caulk the modern Tilia Alsie [7]. Birch pitch was also available in areas with deciduous forests, and was widely traded during the Bronze Age [33], but may have been too brittle to use in caulking boats [34]. As large amounts of pitch would be required to caulk a Hjortspring like boat (up to 6 kg, more than can be provided by a single tree in a year), we argue that this evidence suggests the vessel most likely came from a region where pine trees were in relative abundance.

The southern Baltic region is one of several possible areas that had previously been suggested as a source location for the boat based on the analysis of the material culture found in the vessel [5]. Wooden containers from the boat have their closest stylistic parallels with ceramic vessel finds on both Bornholm and Gotland [5]. These similarities had previously led Kaul (2003:177) to suggest Bornholm as a possible source location for the raiders, a possibility that is supported by our results. The wood containers from the Hjortspring boat also have parallels with ceramics from the Hamburg region, which led Randsborg [16] to suggest northwestern Germany as a possible source location for the attacking army. As the Hamburg region would also have been largely devoid of coniferous trees during the 4^th^ and 3^rd^ centuries BCE, our analysis indicates that an origin in the Baltic region is more likely.

If the Hjortspring boat originated on Bornholm or another coastal location in the Baltic Sea east of Rügen and Scania it would mean that its crew and the rest of their armada traveled over hundreds of kilometers of open water to attack the island of Als. While this is well within the technical capabilities of the Hjortspring boat itself [8], it implies a high degree of logistical and organizational sophistication on the part of the attackers. An attack on Als from the open Baltic would have necessitated passing through much of the Danish archipelago, bypassing the larger islands of Zealand, Falster, and Lolland. This suggests the attack would have been premeditated and planed, possibly as part of a pan-regional political or military dispute. This adds to growing evidence of organized regional warfare in European prehistory [35–37], perhaps best represented by the massive Bronze Age battlefield of Tollense [38–40]. Our suggestion of a Baltic origin for the Hjortspring attackers indicates that maritime warfare was also organized on a regional level during the Pre-Roman Iron Age.

**Fig 12:**
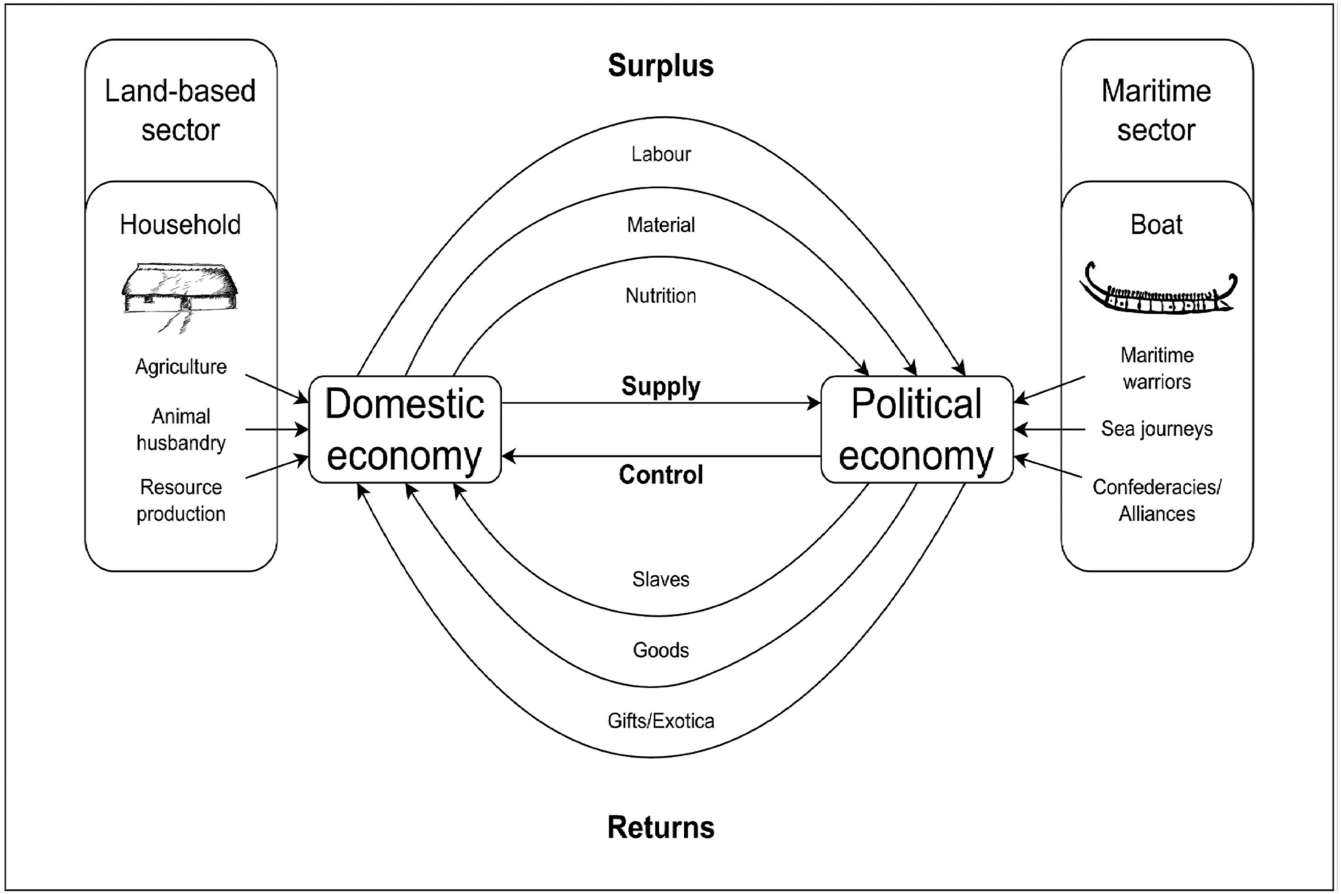
The Maritime Mode of Production as described for the Nordic Bronze Age (from Horn et al. 2024). Evidence that the Hjortspring boat may have been a long-distance maritime attack rather than a local conflict suggests that a similar political economy may have been at work during the Pre-Roman Iron Age.

Long-distance maritime raiding is a characteristic of the Maritime Mode of Production, which has been recently used to explain the organization of both Bronze Age and Viking Age society in Scandinavia [31,33,41]. Under this model, a land-based agropastoral domestic economy exists in a positive-feedback relationship with a maritime-based political economy dependant on trading, raiding, and long-distance political alliances (Fig 12). The maintenance of political networks and regional alliances is key to this model as local leaders would have depended on such systems to navigate regional bottlenecks and to supply trading expeditions. While the Iron Age has previously been characterized as a period in which these long-distance trade and political networks broke down due to the local availability of iron compared to copper and tin, evidence of long-distance targeted raiding suggests that some of these regional alliances and connections were still in existence by at least the 4^rd^ century BCE. Indeed, the breakdown of Bronze Age trading systems may have placed stress on political networks during the early Iron Age leading to increased inter-regional conflict. Hopefully future research will test the hypotheses presented here in order to advance our understanding of Pre-Roman Iron Age political organization.

## Conclusion

Our analysis of the Hjortspring boat cordage and caulking materials using new scientific methods has addressed several long-standing questions regarding the nature of the ancient boat. Our carbon date of the cordage fragment from the original excavation of the vessel confirms its dating to the Pre-Roman Iron Age, most likely during the 4^th^ or early 3^rd^ centuries BCE. Our experimental reconstruction of the cordage also indicates that strings were combined to make both 2 and 4 ply cordage during the sewing of the vessel, answering the question of why some caulking fragments show imprints of cordage with different ply numbers. Our analysis of the cordage fragments has also illuminated the careful and meticulous process involved in retting, picking strands and laying a loose cord optimizing cord strength and pliability.

Our analysis of the boat’s caulking fragments has also advanced our knowledge of the ship’s use and potential provenance. A fingerprint found on a fragment of caulking material also provides us with a direct link to the people who used this ship, although the fact that the print is fragmentary makes it difficult to say more about the individual who left it. Finally, the GC-MS analysis of the caulking material shows that it was made with a mixture of pine pitch and fat, most likely tallow. The use of pine pitch in the ship’s construction and repair suggests that it came from a region rich in pine trees. Based on the dispersal of pine forests in Northern Europe during the first millennium BCE, we suggest that the Hjortspring boat most likely came from somewhere in the Baltic Sea east of the island of Rügen. We propose Blekinge, Bornholm, Gotland, or northern Poland as likely locations and hope that these new results open the door to finally solving the mystery of the boat’s provenance.

Our results also have implications for understanding the social organization of Scandinavia during the Pre-Roman Iron Age. The proposed source locations in the Baltic indicate that raiders who attacked the island of Als chose to launch a maritime raid over hundreds of kilometers of open sea. While it is impossible to know what might have driven them to launch such an attack, the logistics involved in long-distance maritime raids strongly suggest that it was a premeditated and well-organized endeavor. This implies that political disputes in Scandinavia during the Pre-Roman Iron Age eclipsed the local level and may have involved coalitions that spanned multiple regions. Such behavior is highly evocative of the network-oriented maritime modes of production that characterized both the preceding Bronze Age as well as the later Viking Age. Evidence for long-distance maritime raiding and political networks during the Pre-Roman Iron Age indicates that such behavior was a long-standing component of Scandinavian political economies. The Hjortspring find may thus represent a pattern of behavior that characterized Nordic societies for much of their pre-modern history.

## Supporting information

S1 File Experimental Procedures and Data. Experimental Procedures and Data for X-Ray Tomography and GC-MS.
(PDF)
