# Supplementary material for: New investigations of the Hjortspring boat: Dating and analysis of the cordage and caulking materials used in a pre-Roman iron age plank boat
Source: PLoS One. 2025 Dec 10;20(12):e0336965. doi: 10.1371/journal.pone.0336965 (PMC12694875; doi:10.1371/journal.pone.0336965)
Supplement: S1 File — Experimental Procedures and Data for X-Ray Tomography and GC-MS. (PDF) [file pone.0336965.s001.pdf]

Experimental Procedures and Data for X-Ray Tomography and GC-MS

Experimental Procedures for X-Ray Tomography

The X-ray tomography was performed at the 4D imaging Lab at Lund University using an RXSolutions EasyTom150 (Chavanod, France). The X-ray source parameters were 80 kV and 30 W using the medium spot size (approximately 20 microns). For all of the scans, the source-detector distance was 895 mm and each object was placed at an appropriate distance to the source such that the ful sample would fit within the field of view, giving an effective pixel size that was different for each sample and varied between 20-30 microns width for the rope samples and 18.5-57 microns for the tar samples. In each case 1440 radiographic projections were acquired over 360° sample rotation. Tomographic reconstruction was performed with the RXSolutions XAct software (Chavanod, France) to yield image volumes with cubic voxels with the same side length as the effective pixel sizes in the projections. These 3D images were exported as 16 bit tiff slices that could be assembled for 3D visualisation. Data visualisation was performed using the Dragonfly software (Comet Technologies Canada Inc., Montreal, Canada).

The tomography dataset consists of 2D stacks of TIFF images representing the reconstructed 3D volumes of cordage and caulking materials from the Hjortspring boat. The datasets correspond to those shown in Figure 4 of the paper, where the full dataset associated with Figure 4A is available under the title *Rope3*, Figure 4B under *Rope5*, Figure 4C under *TarA688B*, and Figure 4D under *TarA688O*. All files are provided within the Zenodo repository at https://zenodo.org/records/17416700. Models for fingerprint scan in Fig 8 are available on request. Contact Steven Hall at the X-Ray tomography lab, Lund University.

Experimental Procedures for Gas Chromatography – Mass Spectrometry Analysis

Trimethylsilyl derivatization was carried out by combining dried sample (app. 25 mg) with 10 µl internal standard (0,62 mg/ml deuteropalmitate CAS 39756-30-4 d31 in MTBE (methyltertbutylether)), 70 µl anhydrous pyridine and 70 µl N,O-Bis(trimethylsilyl)trifluor-acetamide with trimethylchlorosilane (BSTFA+TMCS) in a 2 ml GC vial. After mixing the vial is left on a heating block at 70 °C for 60 minutes under a tight lid. After cooling the solution is evaporated to dryness under a stream of nitrogen gas. The condensate is re-dissolved in 0.5 ml n-hexane. Not everything dissolves but centrifugation at 5000 rpm for 5 minutes allowed a clear liquid to be isolated and run on GC-MS.

Derivatization by methylation was carried out by adding Ca. 25 mg sample to a 2 ml GC vial together with 10 µl internal standard (0,62 mg/ml deuteropalmitate CAS 39756-30-4 d31 in MTBE (methyltertbutylether)) and 0.5 ml methanol. After mixing, 25µl 96 % sulfuric acid is added and the closed vial is heated for 2 hours at 70°. The liquid is extracted using 3x500 µl hexane, the hexane phase is isolated in a new GC vial and evaporated to dryness under a stream of nitrogen gas. The remainder is re-dissolved in an appropriate amount of hexane and run on GC-MS.

The GC-MS instrument was a Bruker SCION 456GC-TQMS equipped with a Restek Rtx-5 capillary column (30 m, 0.25 mm ID, 0.25 µm) programmed for a 1 ml min^{-1} helium flow. 1 µl sample was applied on the PTV (Programmed Temperature Vaporization) injector which was held at 64 °C for 0.50 min, raised to 315 °C at 200 °C min^{-1} and held at that temperature for 40 min. The GC oven temperature was held at 64 °C for 0.5 min, then raised to 190°C at 10 °C min^{-1} and then to 315 °C at 4 °C min^{-1} and held at that temperature for 15 min. The EI (electron ionisation) source temperature in the mass spectrometer was 250 °C and the

ionisation potential was -70 eV. The mass spectrometer was operated in the full scan mode from *m*/*z* 48 to *m*/*z* 800.

The raw GC-MS data used to create figure 7 is available on the Zenodo repository at https://doi.org/10.5281/zenodo.17428823
